# The Influence of Exogenous Phenylalanine on the Accumulation of Secondary Metabolites in Agitated Shoot Cultures of *Ruta graveolens* L.

**DOI:** 10.3390/molecules28020727

**Published:** 2023-01-11

**Authors:** Agnieszka Szewczyk, Wojciech Paździora, Halina Ekiert

**Affiliations:** Department of Pharmaceutical Botany, Jagiellonian University, Collegium Medicum 9 Medyczna St., 30-688 Cracow, Poland

**Keywords:** *Ruta graveolens*, in vitro cultures, phenolic acids, catechin, linear furanocoumarins, furoquinoline alkaloids, feeding with phenylalanine

## Abstract

This study aimed to examine the influence of the addition of a precursor (phenylalanine) on the accumulation of secondary metabolites in agitated shoot cultures of *Ruta graveolens.* Cultures were grown on Linsmaier and Skoog (LS) medium, with plant growth regulators (0.1 mg/L α-naphthaleneacetic acid—NAA—and 0.1 mg/L 6-benzylaminopurine—BAP). Phenylalanine was added to the cultures at a concentration of 1.25 g/L after 4 and 5 weeks of growth cycles. Biomass was collected after 2, 4, and 7 days of precursor addition. Both control and experimental cultures had the same secondary metabolites accumulated. Using the HPLC method, linear furanocoumarins (bergapten, isoimperatorin, isopimpinellin, psoralen, and xanthotoxin), furoquinoline alkaloids (γ-fagarine, 7-isopentenyloxy-γ-fagarine, and skimmianine), and catechin were detected and quantified in the methanolic extracts. In turn, phenolic acids, such as gallic acid, protocatechuic acid, *p*-hydroxybenzoic acid, syringic acid, *p*-coumaric acid, and ferulic acid were detected in hydrolysates. The production of phenolic acids and catechin (1.5-fold) was significantly increased by the addition of precursor, while there was no significant effect on the production of coumarins and alkaloids. The highest total content of phenolic acids (109 mg/100 g DW) was obtained on the second day of phenylalanine addition (the fourth week of growth cycles). The dominant phenolic compounds were *p*-coumaric acid (maximum content 64.3 mg/100 g DW) and ferulic acid (maximum content 35.6 mg/100 g DW). In the case of catechins, the highest total content (66 mg/100 g DW) was obtained on the third day of precursor addition (the fourth week of growth cycles). This study is the first to document the effect of feeding the culture medium with phenylalanine on the accumulation of bioactive metabolites in in vitro cultures of *R. graveolens*.

## 1. Introduction

Common rue (*Ruta graveolens* L.), belonging to the Rutaceae family, is a species naturally occurring in the Mediterranean region [1]. The rue herb has a very rich chemical composition, which mostly includes alkaloids, flavonoids, phenolic acids, coumarins, and essential oil [2,3,4,5] and, thus, has several healing properties. *Ruta graveolens* also exhibits a wide range of biological activities, such as antioxidant, anti-inflammatory, spasmolytic, sedative, antibacterial, antifungal, and antidiabetic effects [6]. Furthermore, it is a valuable source of some linear furanocoumarins (xanthotoxin, bergapten), which are used in the treatment of skin diseases, such as psoriasis and vitiligo [7,8].

In vitro cultures of *R. graveolens* are a convenient material for investigating the possibilities of increasing the production of secondary metabolites in comparison to the parent plant (grown in soil). So far, different groups of secondary metabolites have been isolated from the biomass cultivated in vitro. These metabolites include essential oil, alkaloids, coumarins, and phenolic acids found in the parent plant, as well as some new compounds that have not been found in the herb [9,10].

Phenolic acids mainly exhibit antioxidant, anti-inflammatory, antimicrobial, choleretic, and immunostimulatory effects [11]. Our previous study on agitated cultures of *R. graveolens* proved their high biosynthetic potential in terms of the production of linear furanocoumarins and furoquinoline alkaloids. However, the content of phenolic compounds produced, including catechin and phenolic acids, was much lower [12]. Therefore, the present study aimed to increase the production of phenolic acids using a special strategy—feeding with phenylalanine, a precursor of the metabolic pathway of this group of compounds. Additionally, the study analyzed the effect of phenylalanine on the accumulation of other bioactive compounds, namely furanocoumarins and furoquinoline alkaloids. Feeding with phenylalanine in in vitro cultures has often resulted in high production of phenolic compounds [13,14,15,16]. Phenylalanine is a basic precursor of different phenolic metabolites in plants. Conversion of this aromatic amino acid via shikimate pathway gives rise to hydroxycinnamic acids [17,18] and, after shortening of hydroxycinnamic acid side chains, results in the formation of hydroxybenzoic acids. Cinnamic acid is the substrate of many metabolic pathways associated with the formation of phenolic acids, coumarins, flavonoids, tannins, anthocyanins, lignans, and spermidine derivatives [19,20]. In this study, for the first time, the shoot cultures of *R. graveolens* were used as material to determine the effect of phenylalanine addition on the production of phenolic compounds (phenolic acids and catechins) and other bioactive metabolites (linear furanocoumarins). The study also investigated the effect of phenylalanine feeding on the production of bioactive furoquinoline alkaloids, as precursor supplementation may have a negative impact on the biosynthesis of other metabolites.

## 2. Results and Discussion

### 2.1. Dynamics of Control and Experimental Culture Growth

The cultures of *R. graveolens* grew in the form of dark green microshoots. During the 4-week growth cycle, a very good increase in dry biomass ranging from 32- to 30-fold was observed in experimental (PheAla) cultures from 2 to 7 days after addition of the precursor. In the case of control cultures, the biomass increase was maintained in the range of 30- to 31-fold from 2 to 7 days after addition of the precursor. In experimental cultures, the dry weight varied from 2.684 to 2.527 g from 2 to 7 days after addition of the precursor, while in control cultures it varied from 2.533 to 2.611 g (Table 1). During the 5-week breeding cycle, the biomass increase in experimental cultures gradually declined and ranged from 30- to 25.1-fold from 2 to 7 days after addition of the precursor. A similar trend was observed in control cultures, in which the biomass increase ranged from 28.6- to 25.2-fold from 2 to 7 days after addition of the precursor. The dry weight in experimental cultures varied from 2.524 to 2.123 g from 2 to 7 days after addition of the precursor. In control cultures, the dry weight varied from 2.416 to 2.132 g from 2 to 7 days after addition of the precursor (Table 1). Comparing the cultivation time, the fourth week was more optimal in terms of the obtained dry mass than the fifth week. The addition of phenylalanine had no significant impact on the growth of cultures, which means that at the applied concentration the precursor did not inhibit the growth of biomass or cause cell damage. These results agree with the findings from the studies of other authors, examining the effect of phenylalanine feeding on the accumulation of secondary metabolites in in vitro cultures of other plant species. In the study on the accumulation of bioactive metabolites in agitated cultures of *Scutellaria lateriflora*, authors used various concentrations of the precursor PheAla (1–2.5 g/L) and found that the optimal concentration of phenylalanine that did not cause inhibition of biomass growth was 1.5 g/L [15]. Skrzypczak-Pietraszek E. et al. confirmed that a phenylalanine concentration of 1.6 g/L was the most advantageous in agitated cultures of *Vitex agnus castus* [16]. On the other hand, Szopa et al. proved that, at concentrations in the range of 0.0165–0.165 g/L, phenylalanine did not inhibit the increase in the biomass of agitated cultures of two *Aronia* spp., while, at higher concentrations (0.825–1.65 g/L), it inhibited biomass growth [13].

A wide range of increments in biomass has been shown in previous studies on different types of in vitro cultures of *R. graveolens*. For example, Ekiert et al. (2001) observed a 5-fold increase in dry biomass in stationary liquid cultures of *R. graveolens* on Linsmaier and Skoog (LS) medium containing naphthaleneacetic acid (NAA; 2 mg/L) and 6-benzylaminopurine (BAP; 2 mg/L). The highest increase in biomass was noted between the 7th and 21st day of cultivation [21]. In subsequent studies in agitated cultures of *R. graveolens* grown on LS medium containing NAA (2 mg/L) and BAP (2 mg/L), a 3.2-fold increase in dry biomass was observed in a 42-day (six-week) breeding cycle [22]. In another study on the agitated cultures of *R. graveolens* grown on LS medium with NAA/BAP at a concentration of 0.1/0.1 mg/L, the highest (33.3-fold) increase in dry biomass was observed in the culture grown for 4 weeks [12]. The differences in biomass growth can be due to differences in inoculum weight and the ratio between inoculum weight and medium volume, lighting conditions, medium composition (plant growth regulators, concentration of vitamin B1), and types of cultures.

### 2.2. Effect of Phenylalanine on the Accumulation of Secondary Metabolites

The HPLC analyses of methanol extracts from the biomass of in vitro cultures (both experimental and control cultures) confirmed the presence of linear furanocoumarins (bergapten, isoimperatorin, isopimpinellin, psoralen, and xanthotoxin), furoquinoline alkaloids (γ-fagarine, 7-isopentenyloxy-γ-fagarine, and skimmianine), and catechin. In turn, the analyses of hydrolysates showed the presence of phenolic acids (gallic acid, protocatechuic acid, *p*-hydroxybenzoic acid, syringic acid, *p*-coumaric acid, and ferulic acid). It was observed that feeding with phenylalanine had a significant effect on the production of phenolic acids and catechin. On the other hand, phenylalanine feeding had no significant effect on the accumulation of linear furanocoumarins and did not cause significant inhibition of the production of bioactive furoquinoline alkaloids.

#### 2.2.1. Accumulation of Phenolic Acids

##### Control Cultures

For the 4-week growth cycle, the total content of phenolic acids determined in the extracts from the biomass of control cultures varied from 73.01 to 81.8 mg/100 g DW from 2 to 7 days after addition of the precursor. The content of the two main phenolic acids, *p*-coumaric acid and ferulic acid, ranged from 46.8 to 56.7 mg/100 g DW, and from 25.3 to 18 mg/100 g DW, respectively, from 2 to 7 days after precursor addition. The content of other phenolic acids ranging from 2 to 7 days after precursor addition was lower and varied as follows: from 1.9 to 3.6 mg/100 g DW for protocatechuic acid, from 1.8 to 2.5 mg/100 g DW for *p*-OH-benzoic acid, and from 1 to 1.5 mg/100 g DW for gallic acid. Syringic acid was accumulated at the smallest amount, varying from 0.05 to 0.03 mg/100 g DW from 2 to 7 days after addition of the precursor.

The total content of phenolic acids determined in the extracts from the biomass of control cultures during the 5-week growth cycle was lower in comparison to the 4-week growth cycle and varied from 80.8 to 59.4 mg/100 g DW from 2 to 7 days after addition of the precursor. The content of the two main phenolic acids, *p*-coumaric acid and ferulic acid, ranged from 58.3 to 38.9 mg/100 g DW and from 17.5 to 13.9 mg/100 g DW, respectively, from 2 to 7 days after precursor addition. The content of other phenolic acids ranging from 2 to 7 days after precursor addition varied as follows: from 2.6 to 1.5 mg/100 g DW for protocatechuic acid, from 1.1 to 4.1 mg/100 g DW for *p*-OH-benzoic acid, and from 1.4 to 0.9 mg/100 g DW for gallic acid. The content of syringic acid varied from 0.02 to 0.03 mg/100 g DW from 2 to 7 days after addition of the precursor.

##### Experimental Cultures

For the 4-week growth cycle, the total content of phenolic acids determined in the extracts from the biomass of experimental (PheAla) cultures varied from 109.6 to 100.7 mg/100 g DW from 2 to 7 days after addition of the precursor. The content of the two main phenolic acids, *p*-coumaric acid and ferulic acid, ranged from 64.3 to 64.1 mg/100 g DW, and from 35.6 to 24.8 mg/100 g DW, respectively, from 2 to 7 days after precursor addition. The content of other phenolic acids ranging from 2 to 7 days after precursor addition was lower and varied as follows: from 4.6 to 4.4 mg/100 g DW for protocatechuic acid, from 3.5 to 6.2 mg/100 g DW for *p*-OH-benzoic acid, from 1.5 to 3 mg/100 g DW for gallic acid, and from 0.17 to 0.24 mg/100 g DW for syringic acid.

Similar to the control cultures, the total content of phenolic acids determined in the extracts from the biomass of experimental cultures during the 5-week growth cycle was lower in comparison to the 4-week growth cycle, and varied from 94 to 81.4 mg/100 g DW from 2 to 7 days after addition of the precursor. The content of the two main phenolic acids, *p*-coumaric acid and ferulic acid, ranged from 62.7 to 53.4 mg/100 g DW, and from 22.5 to 16.5 mg/100 g DW, respectively, from 2 to 7 days after precursor addition. The content of other phenolic acids ranging from 2 to 7 days after precursor addition varied as follows: from 3.7 to 1.7 mg/100 g DW for protocatechuic acid, from 2.6 to 8.2 mg/100 g DW for *p*-OH-benzoic acid, from 2.4 to 1.4 mg/100 g DW for gallic acid, and from 0.02 to 0.03 mg/100 g DW for syringic acid (Table 2).

**Figure 1:**
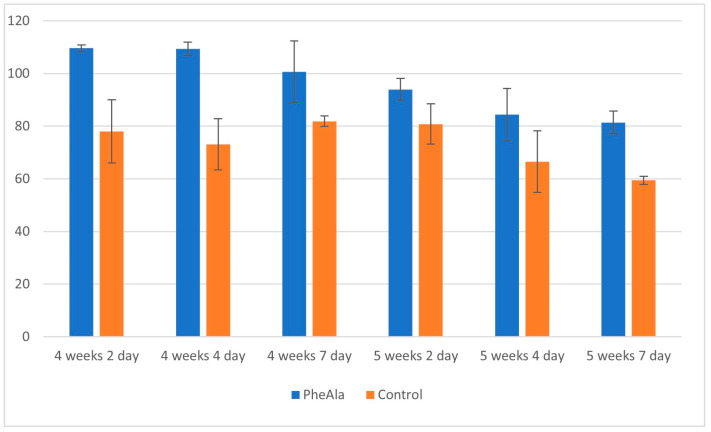
Total content of phenolic acids in the biomass of *R. graveolens* agitated shoot cultures (4- and 5-week growth cycle, time after addition of phenylalanine [PheAla]: 2, 4, and 7 days).

A comparison of the content of phenolic acids in control and experimental (PheAla) cultures showed an increase in the production of these compounds after the addition of the precursor. The total content of phenolic acids in the experimental cultures was higher when compared to the control cultures (4-week growth cycle), and varied from 1.5- to 1.2-fold from 2 to 7 days after precursor addition (Figure 1).

After the 4-week growth cycle, the content of individual phenolic acids in the experimental cultures was found to be higher than that in the control cultures and varied, depending on the day of precursor addition (2, 4, and 7 days), as follows: from 1.4- to 1.1-fold for *p*-coumaric acid, from 1.4- to 1.7-fold for ferulic acid, from 2.3- to 1.2-fold for protocatechuic acid, from 1.4- to 3.4-fold for *p*-OH-benzoic acid, from 1.5- to 2-fold for gallic acid, and from 3.4- to 5.5-fold for syringic acid.

Similarly, for the 5-week growth cycle, an increase of 1.2- to 1.4-fold was observed in the total content of phenolic acids from 2 to 7 days after precursor addition, but the production of most of the individual phenolic acids was lower (Figure 1). The content of individual phenolic acids in the experimental cultures was higher than that in the control, and varied, depending on the day of precursor addition (2, 4, and 7 days), as follows: from 1.1- to 1.3-fold for *p*-coumaric acid, from 1.3- to 1.2-fold for ferulic acid, from 1.5- to 1.1-fold (protocatechuic acid), from 2.5- to 1.9-fold for *p*-OH-benzoic acid, from 1.7- to 1.5-fold for gallic acid, and from 7.8- to 6-fold for syringic acid.

So far, studies on the accumulation of secondary metabolites in *R. graveolens* in vitro cultures have mainly focused on the production of furanocoumarins, while other groups of compounds were less frequently investigated. The qualitative composition of phenolic acids determined in our study is similar to the previously reported results, with some exceptions [23]. This study confirmed the presence of gallic, *p*-hydroxybenzoic, protocatechuic, syringic, *p*-coumaric, and ferulic acids. On the other hand, vanillic acid was not detected, but the content of this compound has been documented in previous studies [23,24]. The quantitative composition of the phenolic acids determined in our study is quite different from that reported in previous studies. In the study by Ekiert et al., protocatechuic acid was shown to be the dominant phenolic acid [23]. On the other hand, in the study by Szopa et al. *p*-coumaric acid was found to be accumulated at the highest amount [24]. In the cultures tested in our study, the main phenolic acids were *p*-coumaric and ferulic acids. It should be emphasized that the individual studies were conducted in different types of cultures with different media composition, under different lighting conditions, which could have caused variations in the qualitative and quantitative composition of the analyzed phenolic acids. It is also important to mention whether the cited authors analyzed the composition of free or bound phenolic acids. For instance, the study by Ekiert et al. [23] determined the content of free phenolic acids in the methanolic extracts of *R. graveolens* shoots grown in stationary liquid cultures. The cultures were maintained on four different variants of the LS medium (with NAA and BAP added at different concentrations, in the range of 0.1–3.0 mg/L). Although the study showed the presence of only four phenolic acids (vanillic, syringic, protocatechuic, and *p*-coumaric) in all the analyzed extracts, the authors also detected other phenolic acids, including *p*-hydroxybenzoic and ferulic acids, in some extracts. The total content of phenolic acids was determined in the range from 85.04 to 108.28 mg/100 g DW (depending on the medium variant). The highest content of the tested phenolic acids was observed on two variants of LS medium: medium with 2 mg/L NAA and 2 mg/L BAP, and medium with 3 mg/L NAA and 1 mg/L BAP [23]. The study by Szopa A. et al. [24] investigated the effect of monochromatic light conditions on the production of free phenolic acids in stationary liquid shoot cultures of *R. graveolens*. The highest content (103.4 mg/100 g DW) of four phenolic acids (protocatechuic acid, syringic acid, *p*-coumaric acid, and vanillic acid) was observed in the biomass from the cultures cultivated on the LS medium, containing 3 mg/L NAA and 1 mg/L BAP under white light. *p*-Coumaric acid and protocatechuic acid were found to be the dominant phenolic acids with maximum content of 43.1 and 37.4 mg/100 g DW, respectively [24]. *p*-Coumaric acid, which is a derivative of cinnamic acid with a phenylhydroxyl group, is the precursor of other phenolic acids, such as caffeic acid, ferulic acid, and chlorogenic acid [25]. In free form, *p*-coumaric acid exhibits a strong antioxidant effect in cells and weakens the process of lipid peroxidation in vivo [26]. Conjugates of *p*-coumaric acid are more commonly found than free *p*-coumaric acid, and are characterized by a very broad biological activity, including antioxidant, anti-inflammatory, antimutagenic, antiulcer, antiplatelet, and anticancer, as well as atherosclerosis-alleviating effect [27]. Ferulic acid has a wide range of potential therapeutic effects, including hepato-, neuro-, and photoprotective effects, and also exhibits antimicrobial and anti-inflammatory activities [28,29].

Based on the origin of the raw material, the total content of phenolic acids in *R. graveolens* herb widely varies from 6.47 to 66.8 mg/100 g DW. The content of protocatechuic and *p*-coumaric acid, which are the main phenolic acids, ranges from 1.34 to 51.9 mg/100 g DW, and from 2.39 to 8.4 mg/100 g DW, respectively [5,23]. The high content of phenolic acids determined in this study indicates that cultures fed with phenylalanine can be a valuable source of phenolic acids comparable to the parent plant.

#### 2.2.2. Accumulation of Catechin

##### Control Cultures

For the 4-week growth cycle, the content of catechin determined in the extracts from the biomass of control cultures varied from 35.7 to 48.4 mg/100 g DW from 2 to 7 days after addition of the precursor. Similarly, for the 5-week growth cycle, the content of catechin determined in the extracts from the biomass of control cultures varied from 39.6 to 43.2 mg/100 g DW from 2 to 7 days after precursor addition.

##### Experimental Cultures

Catechin was also accumulated in greater amounts after feeding with phenylalanine. For the 4-week growth cycle, the content of catechin determined in the extracts from the biomass of experimental (PheAla) cultures varied from 48.9 to 65.9 mg/100 g DW from 2 to 7 days after addition of the precursor. However, the content of catechin determined in the extracts from the biomass of experimental cultures during the 5-week growth cycle was lower in comparison to the 4-week growth cycle, and varied from 53 to 47.7 mg/100 g DW from 2 to 7 days after precursor addition (Table 2).

For the 4-week growth cycle, the content of catechin in the experimental cultures was higher: by 1.4- to 1.3-fold, compared to control cultures ranging from 2 to 7 days after precursor addition. In the case of the 5-week growth cycle, a generally higher increase of 1.3- to 1.1-fold was observed in catechin content after feeding with phenylalanine, ranging from 2 to 7 days after precursor addition, but the production of catechin was lower in comparison to the 4-week growth cycle.

#### 2.2.3. Accumulation of Furanocoumarins

##### Control Cultures

The total content of five linear furanocoumarins determined in the extracts from the biomass of control cultures during the 4-week growth cycle was lower in comparison to the 5-week growth cycle, and varied from 751 to 847 mg/100 g DW from 2 to 7 days after addition of the precursor. The content of other furanocoumarins ranging from 2 to 7 days after precursor addition varied as follows: from 407.7 to 347.8 mg/100 g DW for xanthotoxin, from 156.4 to 307.3 mg/100 g DW for bergapten, from 65.9 to 89.7 mg/100 g DW for isopimpinellin, from 115 to 58.8 mg/100 g DW for psoralen, and from 34.8 to 17 mg/100 g DW for isoimperatorin.

For the 5-week growth cycle, the total content of linear furanocoumarins determined in the extracts from the biomass of control cultures varied from 1059.9 to 907.2 mg/100 g DW from 2 to 7 days after addition of the precursor. The content of other furanocoumarins ranging from 2 to 7 days after precursor addition varied as follows: from 482.5 to 397.1 mg/100 g DW for xanthotoxin, from 330.3 to 351.8 mg/100 g DW for bergapten, from 155.1 to 107.4 mg/100 g DW for isopimpinellin, from 54.6 to 34.7 mg/100 g DW for psoralen, and from 37.4 to 27.4 mg/100 g DW for isoimperatorin.

##### Experimental Cultures

The total content of linear furanocoumarins determined in the extracts from the biomass of experimental (PheAla) cultures during the 4-week growth cycle was lower in comparison to the 5-week growth cycle, and varied from 692 to 755 mg/100 g DW from 2 to 7 days after addition of the precursor. The content of other furanocoumarins ranging from 2 to 7 days after precursor addition varied as follows: from 394.1 to 291.5 mg/100 g DW for xanthotoxin, from 123.5 to 303.4 mg/100 g DW for bergapten, from 57.4 to 85.8 mg/100 g DW for isopimpinellin, from 89.1 to 52.8 mg/100 g DW for psoralen, and from 27.8 to 16.5 mg/100 g DW for isoimperatorin.

For the 5-week growth cycle, the total content of linear furanocoumarins determined in the extracts from the biomass of experimental cultures varied from 931.7 to 827.7 mg/100 g DW from 2 to 7 days after addition of the precursor. The content of other furanocoumarins ranging from 2 to 7 days after precursor addition varied as follows: from 392.3 to 308.2 mg/100 g DW for xanthotoxin, from 345.3 to 392.6 mg/100 g DW for bergapten, from 115.5 to 99.5 mg/100 g DW for isopimpinellin, from 42.4 to 27.9 mg/100 g DW for psoralen), and from 36.2 to 22.8 mg/100 g DW for isoimperatorin (Table 3).

**Figure 2:**
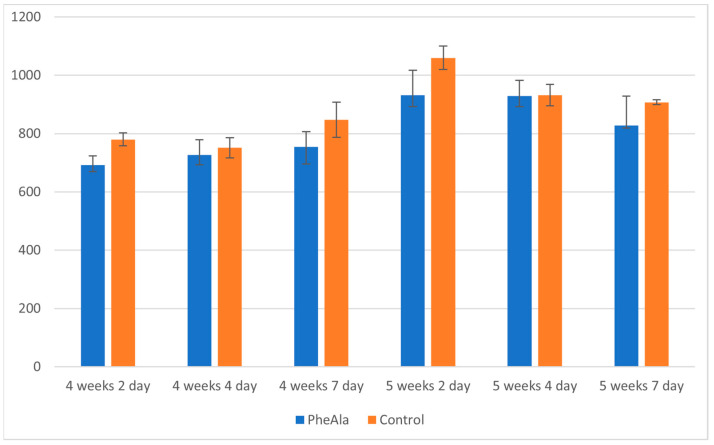
Total content of furanocoumarins in the biomass of *R. graveolens* agitated shoot cultures (4- and 5-week growth cycle, time after addition of phenylalanine [PheAla]: 2, 4, and 7 days).

The unambiguous effect of phenylalanine feeding on the increase in the production of furanocoumarins could not be determined. However, based on the obtained results, it can be concluded that the content of furanocoumarins did not differ much between control and experimental cultures, although the content in control cultures was slightly higher (Figure 2). The precursor may have been mainly utilized in the metabolic pathway of phenolic compounds (particularly cinnamate pathway), whereas, due to the very high degree of accumulation of furanocoumarins, the cyclization of 4-coumaroyl moiety did not occur at an increased rate.

There are many reports on the qualitative and quantitative composition of linear furanocoumarins in *R. graveolens* in vitro cultures. Some of them were also from our laboratory. The authors studied the effect of different types of cultures, different cultivation times, and different compositions and proportions of plant growth regulators. They also used various strategies, such as different light conditions and elicitations, to increase the production of this group of metabolites. Ekiert et al. [21] examined the level of accumulation of furanocoumarins in stationary liquid shoot cultures. Their study confirmed the presence of the following coumarins: psoralen, xanthotoxin, isopimpinellin, bergapten, imperatorin, and umbelliferon. The maximum content of coumarins determined was 966 mg/100 g DW, and the dominant metabolites were found to be xanthotoxin (330 mg/100 g DW) and bergapten (320 mg/100 g DM). The maximum content was achieved after a 4-week growth cycle in LS medium containing 2/2 mg/L NAA/BAP [21]. In subsequent studies performed in stationary liquid cultures maintained under various light conditions on LS medium containing 2/2 mg/L NAA/BAP (6-week breeding cycle), the presence of the five previously mentioned coumarins, as well as umbelliferon, was noted. The highest total content of coumarins (1022.2 mg/100 g DM) was observed in the cultures grown under white constant artificial light. The maximum content of the main furanocoumarins—xanthotoxin and bergapten—was 433.4 and 219.5 mg/100 g DM, respectively [30]. In further studies (Ekiert H., Czygan F.C., 2005), conducted in agitated cultures of *R. graveolens*, it was again observed that the main metabolites were xanthotoxin (maximum content 136.8 mg/100 g DW) and bergapten (maximum content 210.4 mg/100 g DW), and the total content of coumarins after a 6-week growth cycle was 520.8 mg/100 g DW in LS medium containing 0.1/0.1 mg/L NAA/BAP [22]. Similar to previous studies, in the study (Szewczyk A. et al., 2022 [13]) on *R. graveolens* agitated cultures, it was observed that the most dominant coumarins were xanthotoxin (428.3 mg/100 g DW) and bergapten (186.6 mg/100 g DW). The presence of isoimperatorin, isopimpinellin, and psoralen was also confirmed. The maximum total content (917.2 mg/100 g DW) of linear furanocoumarins was reached after a 5-week growth cycle on LS medium containing 0.1/0.1 mg/L NAA/BAP [13]. 

One strategy used to increase the accumulation of secondary metabolites in in vitro cultures is elicitation. The effect of abiotic elicitors (benzothiazole—BTH—and saccharin) was studied in agitated shoot cultures of *R. graveolens* using B_5_ medium (4-week growth cycle). It was observed that the addition of 5% BTH caused an increase in the production of furanocoumarins, such as xanthotoxin (288.36 mg/100 g DW, 8.5-fold increase, compared to control cultures), bergapten (153.78 mg/100 g DW, 3.7-fold increase), isopimpinellin (78.9 mg/100 g DW, 14-fold increase), and that the cultures also produced psoralen (82 mg/100 g DW), which was not found in the control samples [31]. Furthermore, elicitation with another elicitor—chitin (at a concentration of 0.01%)—resulted in a significant increase in the production of xanthotoxin (212 mg/100 g DW, 6.3-fold increase, compared to control cultures), bergapten (146 mg/100 g DW, 3.5-fold increase), isopimpinellin (61 mg/100 g DW, 10.9-fold increase), and psoralen (68 mg/100 g DW, not detected in control cultures) [32]. Elicitation with a biotic elicitor—lysate from *Bacillus* sp. (at a concentration of 25%)—also contributed to a significant increase in the production of xanthotoxin (153.15 mg/100 g DW, 5-fold increase, compared to control cultures), bergapten (90.43 mg/100 g DW, 2-fold increase), isopimpinellin (49 mg/100 g DW, 9-fold increase), and psoralen (52.22 mg/100 g DW, not detected in control cultures) [33].

Among coumarins, the most abundant were xanthotoxin and bergapten, with a maximum content of 482.5 and 392.6 mg/100 g DW, respectively. The content of both these compounds was very high, which was comparable to, or higher than, in the parent plant. For comparison, the content of xanthotoxin and bergapten in soil-grown plants cultivated in Nancy (France) was 410 and 110 mg/100 g DW, respectively [34]. Among the plants growing in the Medicinal Plants Garden of the Jagiellonian University Medical College and the Botanical Garden of the Jagiellonian University in Krakow (Poland), the content of xanthotoxin and bergapten determined in the above-ground parts ranged from 230.4 to 444.1 mg/100 g DW, and from 0 to 39.4 mg/100 g DW, respectively [30]. Furanocoumarins are applied in the so-called PUVA therapy, due to their photosensitizing properties; in particular, bergapten and xanthotoxin are mainly used for this purpose, with the former being better tolerated by patients [7,8].

#### 2.2.4. Accumulation of Furoquinolic Alkaloids

##### Control Cultures

The total content of the three alkaloids determined in the extracts from the biomass of control cultures during the 4-week growth cycle was lower in comparison to the 5-week growth cycle, and varied from 128.7 to 143.3 mg/100 g DW from 2 to 7 days after addition of the precursor. The content of other alkaloids ranging from 2 to 7 days after precursor addition varied as follows: from 57.7 to 69.6 mg/100 g DW for skimmianine, from 55.3 to 68.1 mg/100 g DW for γ-fagarine, and from 5.8 to 4.1 mg/100 g DW for 7-isopentenyloxy-γ-fagarine.

For the 5-week growth cycle, the total content of furoquinolic alkaloids in the extracts from the biomass of control cultures varied from 185.6 to 133 mg/100 g DW from 2 to 7 days after addition of the precursor. The content of other alkaloids ranging from 2 to 7 days after precursor addition varied as follows: from 101.4 to 55.4 mg/100 g DW for skimmianine, from 74.8 to 72.1 mg/100 g DW for γ-fagarine, and from 9.4 to 5.5 mg/100 g DW for 7-isopentenyloxy-γ-fagarine.

##### Experimental Cultures

For the 4-week growth cycle, the total content of furoquinolic alkaloids determined in the extracts from the biomass of experimental cultures varied from 128.2 to 156.1 mg/100 g DW from 2 to 7 days after addition of the precursor. The content of other alkaloids ranging from 2 to 7 days after precursor addition varied as follows: from 57 to 63.6 mg/100 g DW for skimmianine, from 64.8 to 85.4 mg/100 g DW for γ-fagarine, and from 5 to 7.1 mg/100 g DW for 7-isopentenyloxy-γ-fagarine.

For the 5-week growth cycle, the total content of furoquinolic alkaloids determined in the extracts from the biomass of experimental cultures varied from 173.9 to 125.7 mg/100 g DW from 2 to 7 days after addition of the precursor. The content of other alkaloids ranging from 2 to 7 days after precursor addition varied as follows: from 86.5 to 34.3 mg/100 g DW for skimmianine, from 77.6 to 94.5 mg/100 g DW for γ-fagarine, and from 10 to 4.1 mg/100 g DW for 7-isopentenyloxy-γ-fagarine (Table 4).

**Figure 3:**
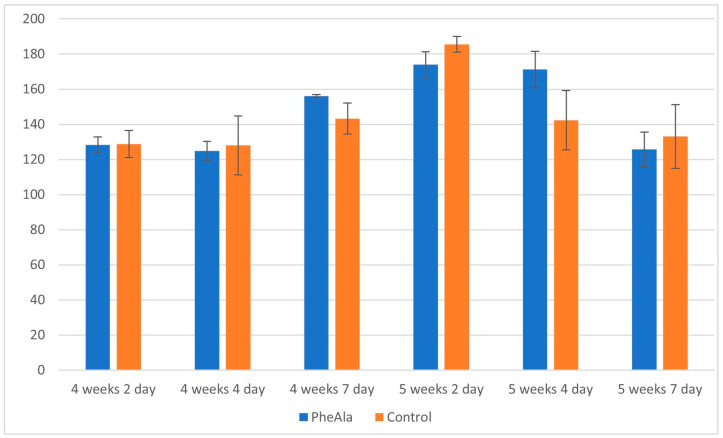
Total content of alkaloids in the biomass of *R. graveolens* agitated shoot cultures (4- and 5-week growth cycle, time after addition of phenylalanine [PheAla]: 2, 4, and 7 days).

In both control and experimental cultures, the total content of the analyzed alkaloids reached the maximum after a 5-week/2-day growth cycle, and then the content began to decrease. No significant inhibition of alkaloid production was observed after phenylalanine feeding (Figure 3).

Among the alkaloids, skimmianine was the most dominant with a maximum content of 101.4 mg/100 g DW (control, 5 weeks, 2 days). γ-Fagarine was also accumulated at large amounts, with the maximum content of 94.5 mg/100 g DW observed after a 5-week/4-day breeding cycle. The in vitro cultures of *R. graveolens* can also be a potential material for the production of furoquinoline alkaloids. Furoquinoline alkaloids show a wide range of biological activities, including antifungal and antibacterial properties, and inhibitory effect against AchE (acetylcholinesterase) enzyme and 5-HT2 receptor [35].

Furoquinoline alkaloids were less frequently studied than linear furanocoumarins in the in vitro cultures of *R. graveolens*. Ekiert H. and Kisiel W. (1997) isolated two alkaloids—kokusaginine and skimmianine—from stationary liquid shoot cultures using spectral methods, and also confirmed their identity [36]. In another study, the authors examined the effects of abiotic elicitors (BTH and saccharin) in agitated shoot cultures of *R. graveolens* using B_5_ medium (4-week growth cycle). They noted that the addition of 5% BTH caused an increase in the production of three alkaloids: γ-fagarine (5.85 mg/100 g DW, 12-fold increase, compared to control cultures), kokusaginine (2.82 mg/100 g DW, 5.3-fold increase), and skimmianine (6.45 mg/100 g DW, 15.7-fold increase) [31]. Furthermore, elicitation with another elicitor—chitin (at a concentration of 0.01%)—also resulted in a significant increase in the production of γ-fagarine (12.65 mg/100 g DW, 36-fold increase), kokusaginine (4.41 mg/100 g DW, 9-fold increase), and skimmianine (14.71 mg/100 g DW, 25-fold increase). In addition, the cultures produced dictamnine (1.04 mg/100 g DW), which was not found in the control samples [32]. Elicitation with a biotic elicitor—lysate from *Pectobacterium atrosepticum* (at a concentration of 1%)—also contributed to a significant increase in the production of γ-fagarine (68.0 mg/100 g DW), kokusaginine (17.2 mg/100 g DW), skimmianine (48.0 mg/100 g DW), and dictamnine (9.9 mg/100 g DW, not detected in control cultures). In control cultures, the content of γ-fagarine and kokusaginine was determined at 0.5 mg/100 g DW, and skimmianine at 0.4 mg/100 g DW [33].

In the study by Szewczyk A. et al. [13] on *R. graveolens* agitated cultures, the dominant furoquinolic alkaloids were also skimmianine (94.6 mg/100 g DW) and γ-fagarine (54.5 mg/100 g DW). The maximum total content (155.9 mg/100 g DW) of these alkaloids was reached after a 5-week growth cycle on LS medium containing 0.1/0.1 mg/L NAA/BAP [13].

#### 2.2.5. The Influence of Feeding with Phenylalanine

Our study showed that phenylalanine feeding can stimulate the production of phenolic acids and catechin in shoot cultures of *R. graveolens*. It should also be emphasized that at the applied concentration (1.25 g/L) the precursor did not inhibit biomass growth. The level of production of other bioactive compounds in the experimental cultures was similar to that in the control cultures. Numerous studies have demonstrated that the production of phenolic compounds in in vitro cultures of different plant species can be stimulated by the phenylalanine feeding method. These studies used cultures of various degrees of differentiation, such as callus cultures, cell suspension cultures, and shoot cultures. For example, in a study on cell suspension cultures of *Ginkgo biloba*, an increase in the production of phenolic acids was observed after the administration of 0.66–1.33 g/L of phenylalanine [14]. In turn, in a study on *Camellia sinensis* callus cultures, the addition of 0.3 g/L of phenylalanine resulted in increased production of catechins [37]. In a study on *Rhodiola imbricata* cell suspension cultures, the addition of phenylalanine at different concentrations (0.5–3 mM) caused an increase in the production of rosavin, rosarin, and *p*-coumaric acid [38]. In a study on *V. agnus castus* shoot cultures, the addition of phenylalanine at a dose of 1.6 g/L led to increased production of phenolic acids and flavonoids [16]. In a study of *S. lateriflora* shoot cultures, feeding with phenylalanine at a concentration of 1.5 g/L was found to be the best strategy to increase the production of flavonoids and verbascoside [15]. In turn, in a study on the cultures of two chokeberry species, *Aronia melanocarpa* and *A. arbutifolia*, lower concentrations of the precursor (0.0165–0.165 g/L) stimulated the production of phenolic acids [13].

An interesting aspect of our study is that the HPLC analyses did not show the presence of any of the analyzed flavonoids, including rutoside—a characteristic flavonoid found in the parent plant. It can be assumed that, in the studied in vitro cultures, the metabolic pathways were blocked at certain stages. The phenylpropanoid pathway may have been blocked at the initial stages, which explains the presence of catechin formed at the beginning of the pathway, and the absence of compounds formed at later stages. The metabolism seems to differ from that of parent plants in in vitro cultures. This may result from damage to the enzyme apparatus or inhibition of the expression of certain genes [39].

## 3. Materials and Methods

### 3.1. Chemicals and Solvents

MeOH, chloroform, glacial acetic acid, and hydrochloric acid of analytical grade were purchased from Chempur (Piekary Slaskie, Poland). Water was purified by a Millipore water purification system (Merck, Darmstadt, Germany). MeOH of HPLC grade was purchased from Merck (Darmstadt, Germany). All chemicals were purchased from Sigma-Aldrich (Saint Louis, MO, USA), unless otherwise mentioned.

The HPLC standards were purchased from the following companies: bergapten, imperatorin, xanthotoxin, and psoralen from Roth (Karlsruhe, Germany); caffeic acid, chlorogenic acid, cinnamic acid, ellagic acid, gallic acid, gentizic acid, isoferulic acid, neochlorogenic acid, *o*-coumaric acid, protocatechuic acid, rosmarinic acid, salicylic acid, sinapic acid, syringic acid, apigenin, apigetrin (apigenin 7-glucoside), hyperoside (quercetin 3-O-galactoside), isoquercetin (quercetin 3-O-glucoside), isorhamnetin, kaempferol, luteolin, myricetin, populnin (kaempferol 7-O-glucoside), robinin (kaempferol 3-O-robinoside-7-O-rhamnoside), quercetin, quercitrin (quercetin 3-O-rhamnoside), rhamnetin, rutoside, vitexin, 5,7-dimethoxycoumarin, 4-hydroxy-6-methylcoumarin, 6- methylcoumarin, osthole, and umbelliferone from Sigma-Aldrich (St Louis, MO, USA); *p*-coumaric acid, vanillic acid, ferulic acid, *p*-hydroxybenzoic acid, coumarin, and scopoletin from Fluka (Bucha, Switzerland); caftaric acid, cryptochlorogenic acid, isochlorogenic acid, catechin, epigallocatechin, epicatechin gallate, epicatechin, epigallocatechin gallate, cinaroside (luteolin 7-O-glucoside), osthenol, 4-methylumbelliferone, 4,6-dimethoxy-2H-1-benzopyran-2-one, and skimmianine from ChromaDex (Irvine, CA, USA); 4-O-feruloylquinic acid, apigetrin (apigenin 7-O-glucoside), apigenin 7-O-glucuronide, astragalin (kaempferol 3-O-glucoside), avicularin (quercetin 3-O-α-L-arabinofuranoside), trifolin (kaempferol 3-O-galactoside), isopimpinellin, isoimperatorin, daphnetin 7-methyl ether, rutaretin, daphnetin, osthenol, bergaptol, daphnetin dimethyl ether, γ-fagarine, and 7-isopentenyloxy-γ-fagarine from ChemFaces (Wuhan, China).

### 3.2. In Vitro Cultures

The shoot cultures of *R. graveolens* were grown in the Department of Pharmaceutical Botany Jagiellonian University, Medical College in Cracow. In vitro cultures of *R. graveolens* were initiated from seeds derived from the Botanical Garden at the Purkyny University in Brno (Czech Republic). Seeds were pre-treated with 5% liquid detergent, rinsed with sterile ultrapure water, then surface-sterilized for 5 min with 0.1% HgCl_2_ and rinsed 3 times with sterile ultrapure water. Sterilized seeds were placed on LS [40] agar-solidified medium, without the addition of plant growth and development regulators, and left to germinate. The hypocotyl fragments of the sprouted seedlings were then passaged on fresh LS liquid medium, containing 1.0 mg/L NAA (auxin) and 1.0 mg/L BAP (cytokinin). Young shoots formed on the explants were passaged on fresh media every 8 weeks. The shoot cultures obtained in this way were used to establish experimental agitated cultures.

The control and experimental agitated cultures were maintained in Erlenmeyer flasks (500 ml) in 150 mL of the medium. The initial biomass was 1 g. Cultures were performed on a shaker (Altel) with a rotation frequency of 140/min. All the cultures were maintained in LS medium under artificial light with an intensity of 4 W/m^2^ at 25 ± 2 °C. The medium contained 0.1 mg/L NAA and 0.1 mg/L BAP. After 4 and 5 weeks of cultivation, phenylalanine solution at a concentration of 1.25 g/L medium was added to the culture flasks. Sterile distilled water was added to the controls. Samples were collected 2, 4, and 7 days after the addition of phenylalanine. The fresh mass of shoot cultures was collected at the end of the cultivation period and dried at 38 °C. Three replicates were performed for each time point in both control and phenylalanine-treated cultures. The results were expressed as mean values (*n* = 3) ± standard deviation (SD) (Excel Microsoft 365).

### 3.3. RP-HPLC Analysis

HPLC analysis was performed to determine the content of metabolites in the methanol extracts from biomass (2 h, at the solvent boiling point of 64.7 °C) and in hydrolysates (2 M HCl, 30 min, at the solvent boiling point of 100 °C) obtained from the agitated shoot cultures. RP-HPLC analysis was carried out as previously described [41] on Merck-Hitachi liquid chromatograph (LaChrom Elite, Hitachi, Tokyo, Japan) equipped with a DAD detector L-2455 and Purospher^®^ RP-18e (250 × 4 mm/5 mm) column (Merck, Darmstadt, Germany). Analysis was carried out at 25 °C. The mobile phase used for the analysis consisted of methanol (A), and methanol:0.5% acetic acid (1:4, *v*/*v*) (B). The flow rate was 1 mL/min, and the gradient was as follows: 100% B for 0–20 min; 100–80% B for 20–35 min; 80–60% B for 35–55 min; 60–0% B for 55–70 min; 0% B for 70–75 min; 0–100% B for 75–80 min; and 100% B for 80–90 min. Quantification was done by measuring the peak area with reference to a standard curve derived from five concentrations (0.03125–0.5 mg/mL). Exemplary chromatograms showing the content of the analyzed compounds in an extract from *R. graveolens* cultures and chromatograms of the standards are included in the Supplementary Files (Figures S1–S4).

### 3.4. Statistical Analyses

All statistical analyses were conducted using the STATISTICA 13.3 software program (TIBCO Software Co., Palo Alto, CA, USA). The level of significance was set at *p* < 0.05. The differences in values across the groups were analyzed by a two-way analysis of variance, followed by Tukey’s post hoc test. The results were expressed as mean ± SD. The results of the comparison of homogeneous groups of control cultures and with the addition of phenylalanine are presented in the Supplementary Materials.

## 4. Conclusions

In summary, it can be concluded that the tested biotechnological strategy, i.e., feeding of culture media with phenylalanine, increased the production of phenolic acids and catechin. The level of production of other bioactive metabolites, including linear furanocoumarins and furoquinolic alkaloids, was comparable to that of control cultures. Due to the promising results obtained in this study, in the future, an attempt can be made to increase the production of bioactive compounds in *R. graveolens* cultures, using simultaneously combined methods: elicitation with abiotic or biotic elicitors and feeding with phenylalanine.

## Figures and Tables

**Table 1:** Dry weight [g] obtained from *R. graveolens* agitated cultures. Mean of three replications. Different letters indicate significant differences (*p* < 0.05). PheAla—phenylalanine.

Growth Period		Dry Weight [g]	
Week	Day after Addition of PheAla	PheAla	Control
4	2	2.684 ± 0.025 ^abc^	2.533 ± 0.068 ^abcdef^
4	4	2.595 ± 0.150 ^abcdef^	2.611 ± 0.070 ^abcdf^
4	7	2.527 ± 0.054 ^abcdef^	2.565 ± 0.105 ^abcdef^
5	2	2.524 ± 0.058 ^abcdef^	2.416 ± 0.040 ^bcdef^
5	4	2.350 ± 0.088 ^bcdefgh^	2.389 ± 0.107 ^bcdefh^
5	7	2.123 ± 0.099 ^egh^	2.132 ± 0.116 ^efgh^

^a–h^ The letters represent homogeneous groups.

**Table 2:** Average content of phenolic compounds (phenolic acids, catechin) [mg/100 g DW] in hydrolysates obtained from the biomass of *R. graveolens* agitated cultures, depending on the duration of the growth cycle (4 and 5 weeks) and day after addition of phenylalanine (2, 3, and 7 days). Means of three measurements ± SD. Different letters indicate significant differences (*p* < 0.05). PheAla—phenylalanine.

Metabolite	Growth Period		Content [mg/100 g DW]	
	Week	Day	PheAla	Control
*p*-Coumaric acid	4	2	64.280 ± 2.383 ^ab^	46.822 ± 10.067 ^abc^
	4	4	64.203 ± 4.690 ^ab^	48.956 ± 11.022 ^abc^
	4	7	64.087 ± 12.045 ^ab^	56.736 ± 1.674 ^abc^
	5	2	62.736 ± 1.327 ^ab^	58.277 ± 6.128 ^abc^
	5	4	55.010 ± 6.630 ^abc^	46.716 ± 12.286 ^abc^
	5	7	53.412 ± 4.403 ^abc^	38.927 ± 2.009 ^bc^
Ferulic acid	4	2	35.573 ± 1.935 ^ac^	25.339 ± 2.787 ^bcdeg^
	4	4	31.464 ± 1.557 ^abc^	18.827 ± 3.174 ^bdefghi^
	4	7	24.796 ± 1.532 ^bdegh^	18.014 ± 2.621 ^dfghi^
	5	2	22.515 ± 2.503 ^bdefgh^	17.476 ± 2.887 ^dfghi^
	5	4	18.219 ± 2.067 ^defghi^	14.148 ± 1.589 ^dfhi^
	5	7	16.546 ± 2.572 ^dfghi^	13.903 ± 0.305 ^dfhi^
*p*-OH-benzoic acid	4	2	3.476 ± 0.931 ^abcdefi^	2.532 ± 0.540 ^abefi^
	4	4	6.172 ± 0.931 ^acdghi^	1.834 ± 0.426 ^abefi^
	4	7	5.349 ± 1.414 ^acdegi^	2.326 ± 0.475 ^abefi^
	5	2	2.587 ± 0.652 ^abdefi^	1.081 ± 0.198 ^abef^
	5	4	6.300 ± 1.153 ^cdghi^	2.526 ± 1.168 ^abefi^
	5	7	8.170 ± 1.819 ^cgh^	4.161 ± 0.100 ^abcdegi^
Protocatechuic acid	4	2	4.635 ± 1.201 ^ace^	2.251 ± 0.491 ^bcde^
	4	4	4.440 ± 1.463 ^abce^	1.882 ± 0.438 ^bde^
	4	7	4.515 ± 1.230 ^ace^	3.601 ± 0.525 ^abcde^
	5	2	3.656 ± 0.477 ^abcde^	2.583 ± 0.451 ^abcde^
	5	4	3.305 ± 0.532 ^abcde^	2.190 ± 0.476 ^bde^
	5	7	1.676 ± 0.429 ^bde^	1.472 ± 0.266 ^bde^
Gallic acid	4	2	1.481 ± 0.383 ^abd^	0.988 ± 0.400 ^ab^
	4	4	3.023 ± 0.314 ^cd^	1.483 ± 0.341 ^abd^
	4	7	1.693 ± 0.289 ^abd^	1.119 ± 0.222 ^ab^
	5	2	2.320 ± 0.265 ^acd^	1.371 ± 0.364 ^ab^
	5	4	1.368 ± 0.419 ^ab^	0.864 ± 0.111 ^ab^
	5	7	1.371 ± 0.323 ^ab^	0.914 ± 0.062 ^ab^
Syringic acid	4	2	0.170 ± 0.067 ^abcd^	0.049 ± 0.034 ^abc^
	4	4	0.118 ± 0.021 ^abcd^	0.031 ± 0.016 ^abc^
	4	7	0.240 ± 0.071 ^acd^	0.044 ± 0.029 ^abc^
	5	2	0.141 ± 0.034 ^abcd^	0.018 ± 0.004 ^bc^
	5	4	0.130 ± 0.020 ^abcd^	0.041 ± 0.021 ^abc^
	5	7	0.204 ± 0.126 ^acd^	0.034 ± 0.019 ^abc^
Total phenolic acids	4	2	109.615 ± 1.197 ^ade^	77.982 ± 11.992 ^bcdefg^
	4	4	109.419 ± 2.530 ^ade^	73.013 ± 9.719 ^bcefg^
	4	7	100.681 ± 11.683 ^abde^	81.839 ± 1.981 ^bcdefg^
	5	2	93.957 ± 4.184 ^abcde^	80.807 ± 7.586 ^bcdefg^
	5	4	84.333 ± 9.989 ^bcdef^	66.485 ± 11.688 ^bcefg^
	5	7	81.380 ± 4.282 ^bcdefg^	59.410 ± 1.503 ^bcfg^
Catechin	4	2	48.894 ± 2.140 ^abd^	35.666 ± 5.471 ^ab^
	4	4	65.911 ± 6.213 ^cd^	45.896 ± 5.963 ^abd^
	4	7	65.322 ± 8.856 ^cd^	48.374 ± 5.563 ^abd^
	5	2	51.297 ± 2.323 ^acd^	39.603 ± 1.034 ^abd^
	5	4	52.994 ± 3.067 ^acd^	40.732 ± 2.493 ^abd^
	5	7	47.667 ± 4.010 ^abd^	43.187 ± 7.987 ^abd^

^a–i^ The letters represent homogeneous groups.

**Table 3:** Average content of furanocoumarins [mg/100 g DW] in methanol extracts obtained from the biomass of *R. graveolens* agitated cultures, depending on the duration of the growth cycle (4 and 5 weeks) and day after addition of phenylalanine (2, 3, and 7 days). Means of three measurements ± SD. Different letters indicate significant differences (*p* < 0.05). PheAla—phenylalanine.

Metabolite	Growth Period		Content [mg/100 g DW]	
	Week	Day	PheAla	Control
Xanthotoxin	4	2	394.131 ± 15.658 ^a–j^	407.724 ± 25.454 ^a–i^
	4	4	305.047 ± 15.315 ^acdefhj^	347.801 ± 19.053 ^abcdefhij^
	4	7	291.519 ± 21.246 ^bcdehj^	362.567 ± 20.296 ^abcdefhij^
	5	2	392.355 ± 48.182 ^abcdfhij^	482.470 ± 24.150 ^abdgi^
	5	4	361.163 ± 50.342 ^abcdefhij^	397.132 ± 14.004 ^abdfghij^
	5	7	308.183 ± 53.323 ^acdefhij^	398.559 ± 15.831 ^abdfghi^
Bergapten	4	2	123.491 ± 6.170 ^abd^	156.366 ± 12.296 ^abcd^
	4	4	229.479 ± 48.314 ^bcdeg^	216.975 ± 14.859 ^abcde^
	4	7	303.376 ± 40.991 ^cdefg^	307.338 ± 52.316 ^cdefg^
	5	2	345.300 ± 49.028 ^efg^	330.286 ± 16.630 ^cefg^
	5	4	392.606 ± 39.558 ^efg^	351.777 ± 38.942 ^efg^
	5	7	369.260 ± 46.468 ^efg^	339.258 ± 11.359 ^efg^
Isopimpinellin	4	2	57.371 ± 15.222 ^abc^	65.932 ± 12.874 ^abcd^
	4	4	77.971 ± 6.558 ^abcdh^	84.550 ± 1.399 ^bcdgh^
	4	7	85.765 ± 2.499 ^bcdgh^	89.718 ± 3.069 ^bcdgh^
	5	2	115.485 ± 1.410 ^gh^	155.122 ± 13.326 ^f^
	5	4	108.179 ± 6.982 ^degh^	108.141 ± 8.868 ^degh^
	5	7	99.471 ± 7.609 ^cdegh^	107.366 ± 9.646 ^degh^
Psoralen	4	2	89.095 ± 6.306 ^abc^	115.023 ± 11.123 ^ab^
	4	4	97.699 ± 9.206 ^ab^	84.760 ± 35.761 ^abc^
	4	7	52.828 ± 11.108 ^acd^	58.771 ± 6.269 ^acd^
	5	2	42.372 ± 2.999 ^cd^	54.565 ± 3.980 ^acd^
	5	4	39.010 ± 2.987 ^cd^	46.799 ± 9.714 ^cd^
	5	7	27.906 ± 3.390 ^cd^	34.680 ± 2.350 ^cd^
Isoimperatorin	4	2	27.817 ± 3.217 ^abde^	34.852 ± 2.172 ^abefg^
	4	4	16.435 ± 2.491 ^cd^	16.958 ± 2.710 ^cd^
	4	7	21.480 ± 1.123 ^acd^	28.619 ± 1.515 ^abdef^
	5	2	36.234 ± 2.890 ^befg^	37.424 ± 5.333 ^bfg^
	5	4	27.746 ± 2.793 ^abde^	27.788 ± 1.920 ^abde^
	5	7	22.851 ± 1.514 ^acde^	27.359 ± 1.007 ^abde^
Total furanocoumarins	4	2	691.904 ± 31.895 ^abc^	779.898 ± 22.242 ^abcde^
	4	4	726.631 ± 51.849 ^abc^	751.044 ± 34.123 ^abce^
	4	7	754.967 ± 51.666 ^abce^	847.013 ± 59.924 ^abcde^
	5	2	931.745 ± 85.260 ^bde^	1059.867 ± 39.735 ^de^
	5	4	928.703 ± 53.616 ^bde^	931.637 ± 36.830 ^bde^
	5	7	827.671 ± 100.914 ^abcde^	907.222 ± 8.184 ^bcde^

^a–j^ The letters represent homogeneous groups.

**Table 4:** Average content of furoquinoline alkaloids [mg/100 g DW] in methanol extracts obtained from the biomass of *R. graveolens* agitated cultures, depending on the duration of the growth cycle (4 and 5 weeks) and day after addition of phenylalanine (2, 3, and 7 days). Means of three measurements ± SD. Different letters indicate significant differences (*p* < 0.05). PheAla—phenylalanine.

Metabolite	Growth Period		Content [mg/100 g DW]	
	Week	Day	PheAla	Control
Skimmianine	4	2	57.045 ± 6.269 ^abcf^	67.562 ± 3.212 ^abcdf^
	4	4	50.343 ± 7.391 ^abcg^	57.730 ± 10.786 ^abcf^
	4	7	63.643 ± 5.420 ^abcf^	69.631 3.540 ^abcdf^
	5	2	86.473 ± 6.840 ^bdef^	101.427 ± 4.746 ^de^
	5	4	71.415 ± 5.959 ^abdf^	65.430 ± 10.416 ^abcf^
	5	7	34.319 ± 1.966 ^cg^	55.418 ± 9.433 ^abcf^
γ-Fagarine	4	2	64.779 ± 2.068 ^abcefg^	55.283 ± 4.184 ^abce^
	4	4	69.472 ± 5.161 ^abcefgi^	66.130 ± 8.776 ^abcefg^
	4	7	85.365 ± 5.773 ^cdefghi^	68.100 ± 6.356 ^abcdefg^
	5	2	77.548 ± 1.013 ^acdefghi^	74.775 ± 1.572 ^acdefgi^
	5	4	94.516 ± 4.008 ^dfhi^	72.591 ± 7.443 ^abcdefgi^
	5	7	87.309 ± 8.098 ^cdfghi^	72.116 ± 12.477 ^abcdefgi^
7-Isopentenyloxy-γ-fagarine	4	2	6.413 ± 0.015 ^abce^	5.831 ± 1.114 ^abcde^
	4	4	4.970 ± 1.035 ^abcd^	4.101 ± 0.454 ^bcd^
	4	7	7.085 ± 0.120 ^abe^	5.552 ± 0.223 ^abcde^
	5	2	9.892 ± 0.164 ^f^	9.369 ± 0.242 ^f^
	5	4	5.385 ± 0.683 ^abcde^	4.196 ± 0.185 ^bcd^
	5	7	4.065 ± 1.186 ^bcd^	5.481 ± 0.943 ^abcde^
Total furoquinolic alkaloids	4	2	128.237 ± 4.549 ^abcgh^	128.676 ± 7.718 ^abcgh^
	4	4	124.785 ± 5.537 ^abcgh^	127.962 ± 16.726 ^abcgh^
	4	7	156.094 ± 0.897 ^abcdefgh^	143.284 ± 8.889 ^abcdfgh^
	5	2	173.913 ± 7.439 ^bcdef^	185.571 ± 4.433 ^bdef^
	5	4	171.316 ± 10.184 ^bcdefg^	142.216 ± 16.881 ^abcfgh^
	5	7	125.694 ± 9.875 ^abcgh^	133.015 ± 18.048 ^abcgh^

^a–i^ The letters represent homogeneous groups.

## Data Availability

The data presented in this study are available on request from the corresponding author.

## Supplementary Materials

The following supporting information can be downloaded at: https://www.mdpi.com/article/10.3390/molecules28020727/s1, Figure S1: Sample chromatogram of the extract from *Ruta graveolens* in vitro cultures (0.1/0.1 LS medium, 5-week growth cycle, 2 day after PheAla addition) 1. catechin 2. psoralen, 3. xanthotoxin, 4. isopimpinellin, 5. skimmianine, 6. bergapten, 7. γ-fagarine, 8. isoimperatorin, 9. 7-isopentenyloxy-γ-fagarine.; Figure S2: Sample chromatograms of the standards: 1. catechin 2. psoralen, 3. xanthotoxin, 4. isopimpinellin, 5. skimmianine, 6. bergapten, 7. γ-fagarine, 8. isoimperatorin, 9. 7-isopentenyloxy-γ-fagarine.; Figure S3: Enlarged fragment of a sample chromatogram of the hydrolysate from *R. graveolens* in vitro cultures (0.1/0.1 LS medium, 5-week growth cycle, 2 day after PheAla addition) 1. gallic acid, 2. protocatechuic acid, 3. *p*-hydroxybenzoic acid, 4. syringic acid, 5. *p*-coumaric acid, and 6. ferulic acid.; Figure S4: Sample chromatograms of the standards: 1. gallic acid, 2. protocatechuic acid, 3. *p*-hydroxybenzoic acid, 4. syringic acid, 5. *p*-coumaric acid, and 6. ferulic acid.

## Sample Availability

Samples of the extracts of *R. graveolens* in vitro cultures are available from the authors.
