# Supplementary material for: The Influence of Exogenous Phenylalanine on the Accumulation of Secondary Metabolites in Agitated Shoot Cultures of Ruta graveolens L
Source: Molecules. 2023 Jan 11;28(2):727. doi: 10.3390/molecules28020727 (PMC9864388; doi:10.3390/molecules28020727)
Supplement: Supplementary file 1 [file molecules-28-00727-s001.zip › molecules-2137630-supplementary.pdf]

SUPPLEMENTARY FILES

The influence of exogenous phenylalanine on the accumulation of secondary metabolites in agitated shoot cultures of *Ruta graveolens* L.

I. HPLC ANALYSIS

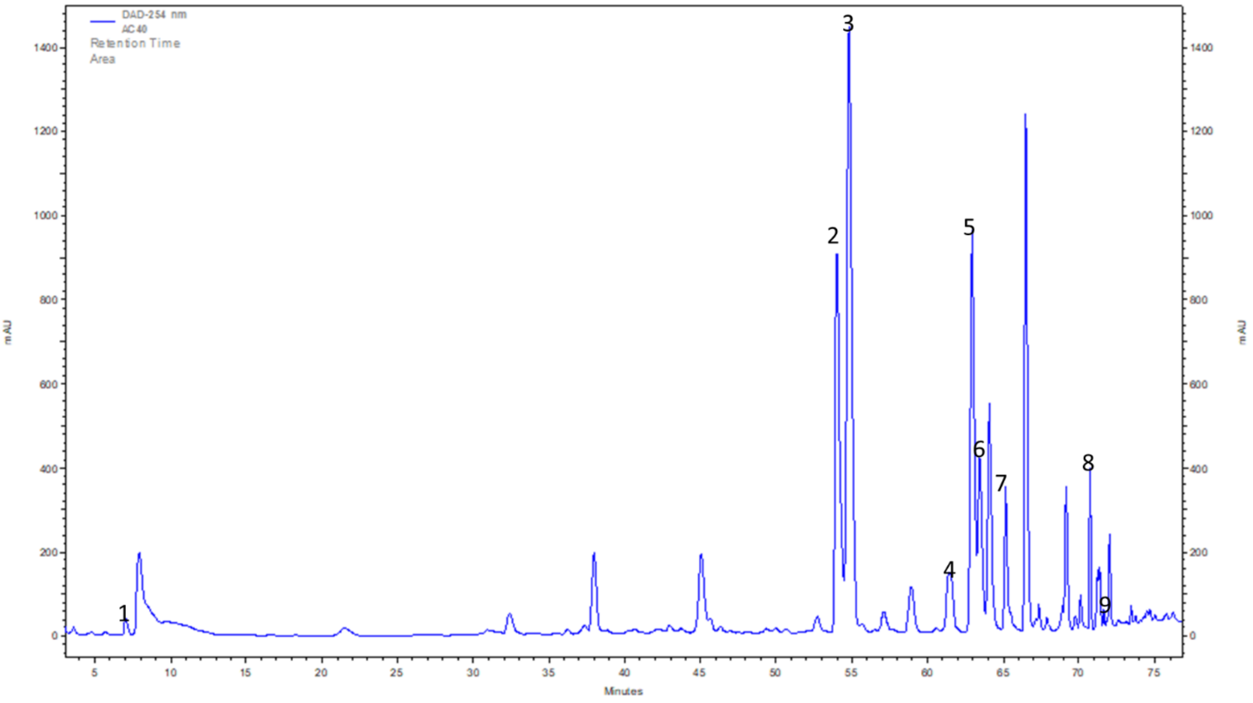

Figure S1 Sample chromatogram of the extract from *Ruta graveolens in vitro* cultures (0.1/0.1 LS medium, 5-week growth cycle, 2 day after PheAla addition) 1. catechin 2. psoralen, 3. xanthotoxin, 4. isopimpinellin, 5. skimmianine, 6. bergapten, 7. γ-fagarine, 8. isoimperatorin, 9. 7-isopentenyloxy-γ-fagarine

catechin

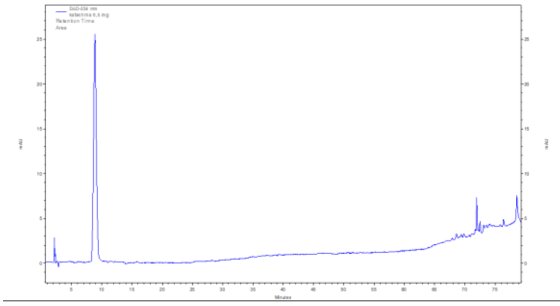

psoralen

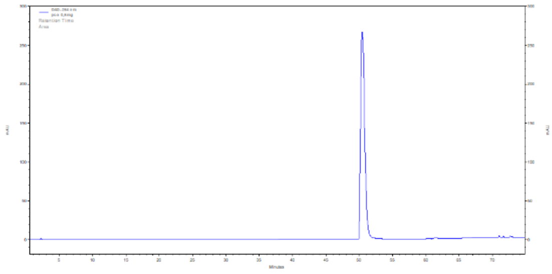

xanthotoxin

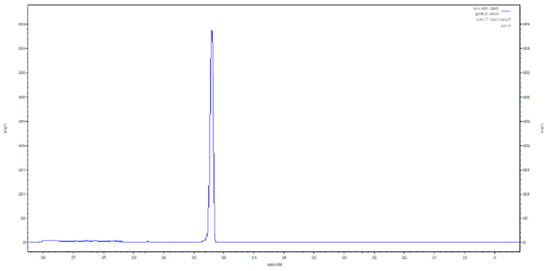

isopimpinellin

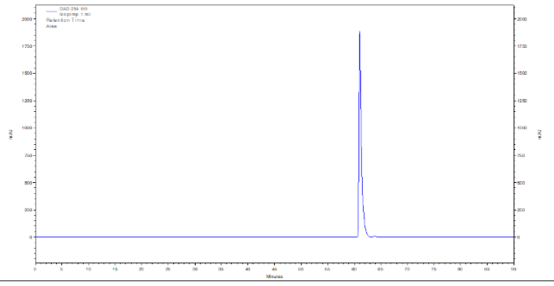

skimmianine

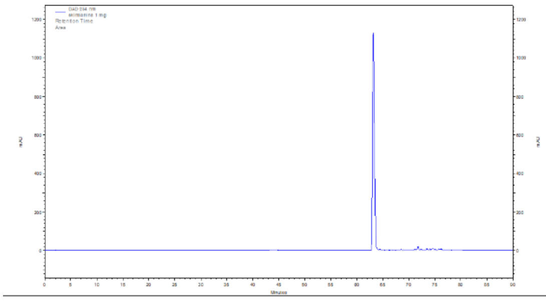

bergapten

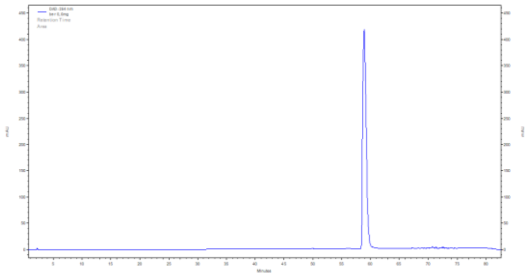

γ-fagarine

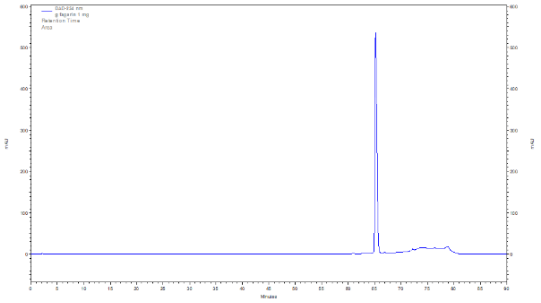

isoimperatorin

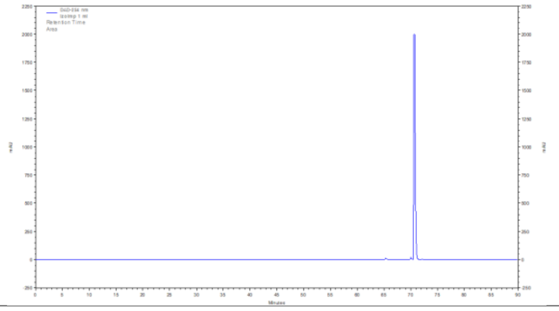

7-isopentenyloxy-γ-fagarine

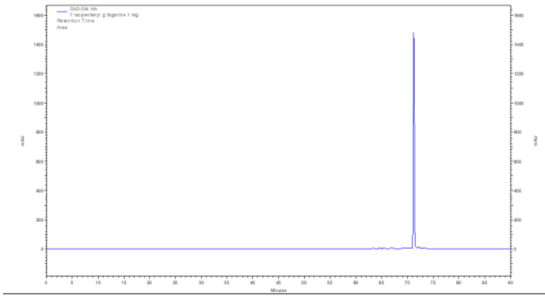

Figure S2 Sample chromatograms of the standards: 1. catechin 2. psoralen, 3. xanthotoxin, 4. isopimpinellin, 5. skimmianine, 6. bergapten, 7. γ-fagarine, 8. isoimperatorin, 9. 7-isopentenyloxy-γ-fagarine

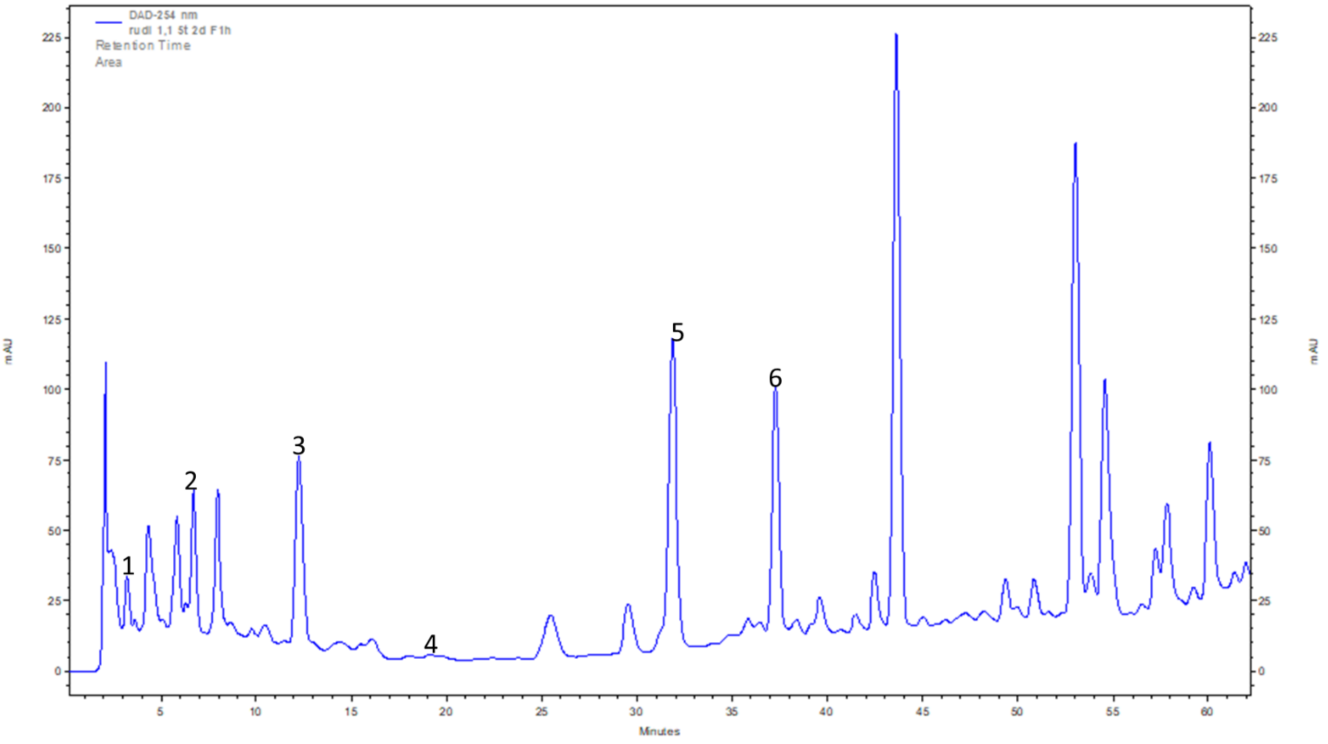

Figure S3 Enlarged fragment of a sample chromatogram of the hydrolysate from *R. graveolens in vitro* cultures (0.1/0.1 LS medium, 5-week growth cycle, 2 day after PheAla addition) 1. gallic acid, 2. protocatechuic acid, 3. *p*-hydroxybenzoic acid, 4. syringic acid, 5. *p*-coumaric acid, and 6. ferulic acid.

gallic acid

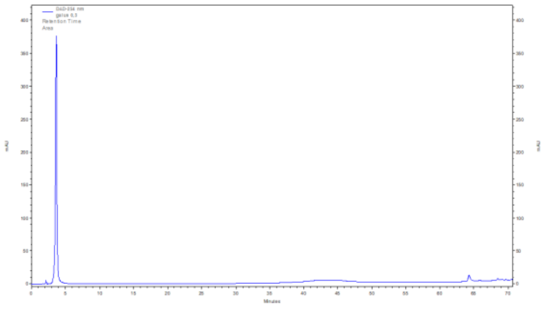

protocatechuic acid

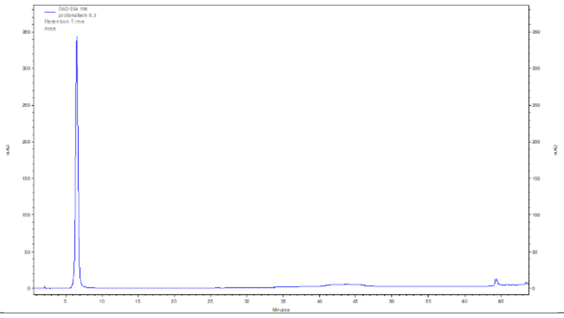

p-hydroxybenzoic acid

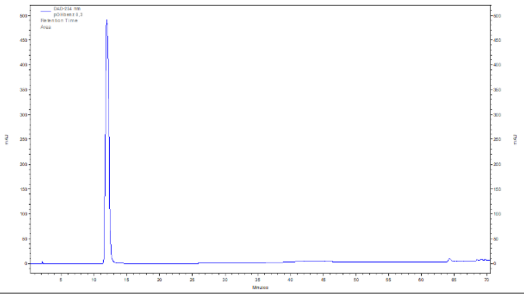

syringic acid

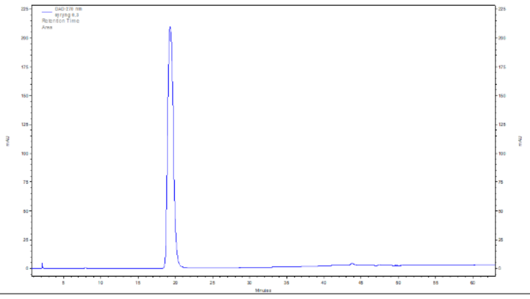

p-coumaric acid

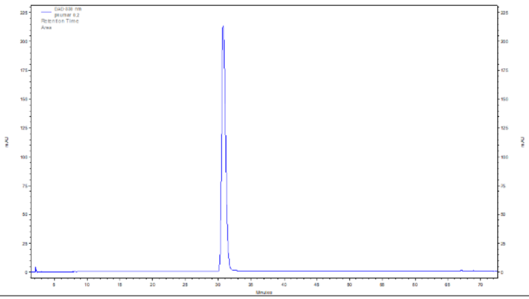

ferulic acid

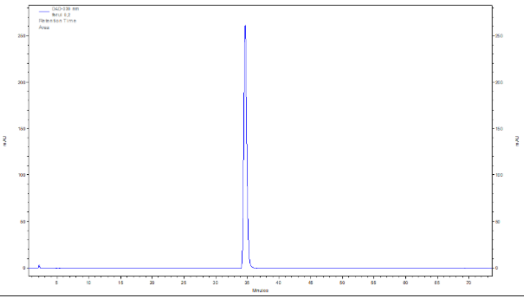

Figure S4 Sample chromatograms of the standards: 1. gallic acid, 2. protocatechuic acid, 3. *p*-hydroxybenzoic acid, 4. syringic acid, 5. *p*-coumaric acid, and 6. ferulic acid.

2. STATISTICAL ANALYSIS

2.1 Comparison of homogeneous groups: control cultures and cultures fed with phenylalanine, in terms of phenolic acid production.

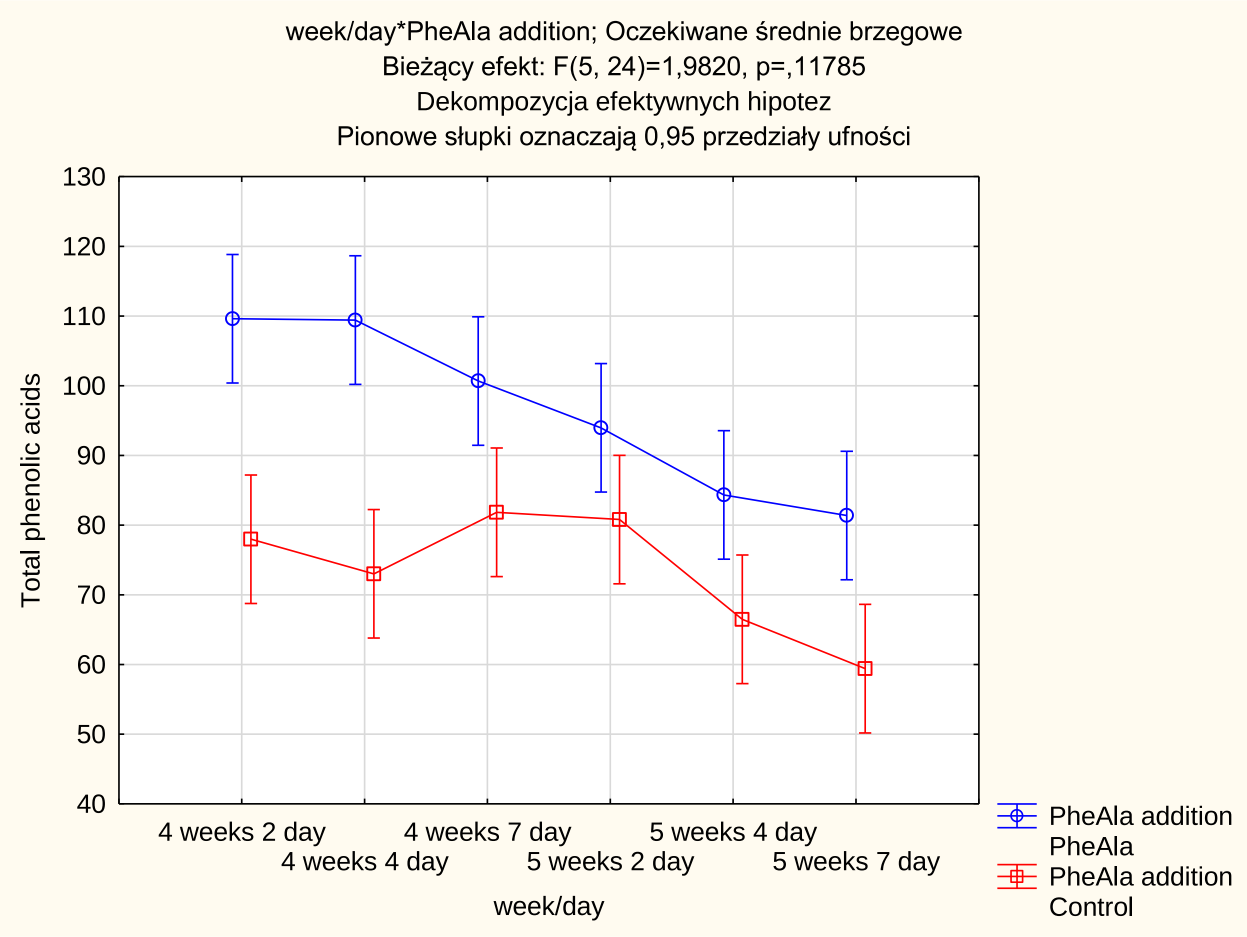

No.	Tukey test; Total phenolic acids; homogenous groups; alpha = 0.05							
	week/day	PheAla addition	Total phenolic acids	1	2	3	4	5
12	5 weeks 7 day	Control	59,4103	****				
10	5 weeks 4 day	Control	66,4850	****	****			
4	4 weeks 4 day	Control	73,0130	****	****	****		
2	4 weeks 2 day	Control	77,9820	****	****	****	****	
8	5 weeks 2 day	Control	80,8067	****	****	****	****	
6	4 weeks 7 day	Control	81,8387	****	****	****	****	
11	5 weeks 7 day	PheAla	81,3803	****	****	****	****	
9	5 weeks 4 day	PheAla	84,3327		****	****	****	
7	5 weeks 2 day	PheAla	93,9567			****	****	****
5	4 weeks 7 day	PheAla	100,6807				****	****
3	4 weeks 4 day	PheAla	109,4193					****
1	4 weeks 2 day	PheAla	109,6146					****

2.2 Comparison of homogeneous groups: control cultures and cultures fed with phenylalanine, in terms of catechin production.

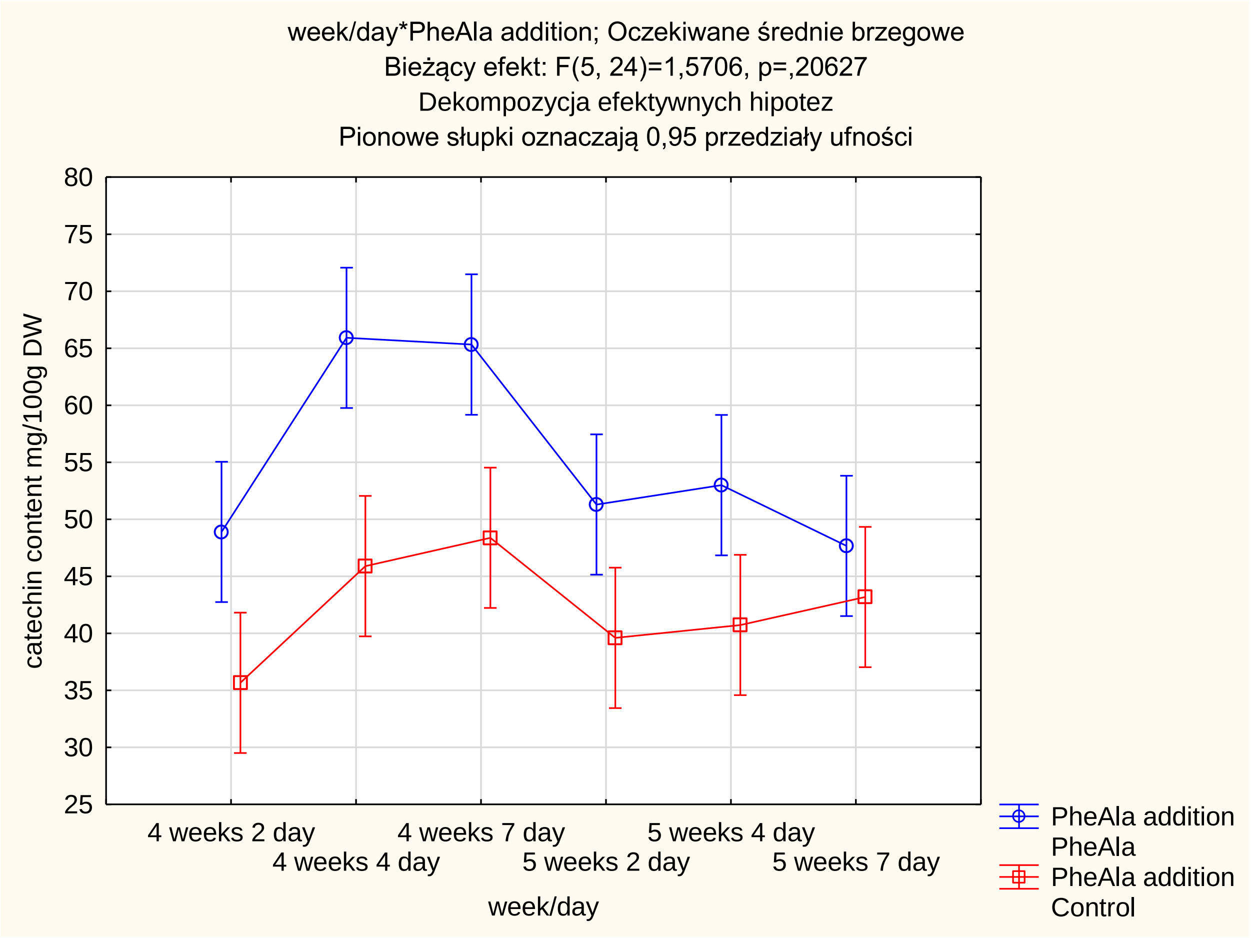

No.	Tukey test; catechin content mg/100g DW; homogenous groups, alpha = 0.05					
	week/day	PheAla addition	catechin content mg/100g DW	1	2	3
2	4 weeks 2 day	Control	35,66600		****	
8	5 weeks 2 day	Control	39,60343	****	****	
10	5 weeks 4 day	Control	40,73167	****	****	
12	5 weeks 7 day	Control	43,18700	****	****	
4	4 weeks 4 day	Control	45,89633	****	****	
6	4 weeks 7 day	Control	48,37367	****	****	
11	5 weeks 7 day	PheAla	47,66700	****	****	
1	4 weeks 2 day	PheAla	48,89433	****	****	
7	5 weeks 2 day	PheAla	51,29733	****		****
9	5 weeks 4 day	PheAla	52,99433	****		****
5	4 weeks 7 day	PheAla	65,32233			****
3	4 weeks 4 day	PheAla	65,91067			****

2.3 Comparison of homogeneous groups: control cultures and cultures fed with phenylalanine, in terms of furoquinolic alkaloids production.

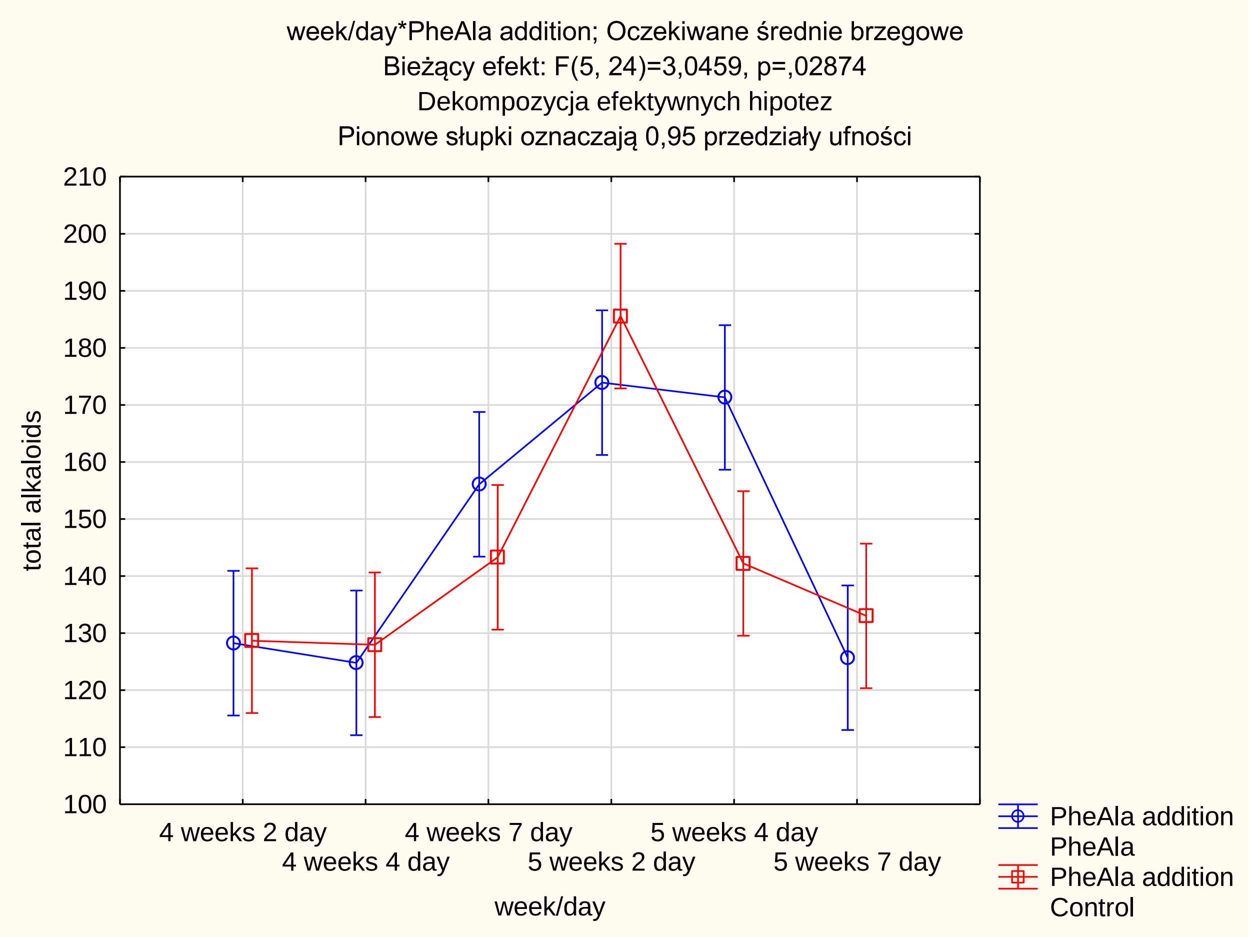

Nr podkl.	Tukey test; total alkaloids; homogenous goups, alpha = 0.05						
	week/day	PheAla addition	total alkaloids	1	2	3	4
3	4 weeks 4 day	PheAla	124,7853	****			
11	5 weeks 7 day	PheAla	125,6937	****			
4	4 weeks 4 day	Control	127,9617	****			
1	4 weeks 2 day	PheAla	128,2367	****			
2	4 weeks 2 day	Control	128,6757	****			
12	5 weeks 7 day	Control	133,0153	****			
10	5 weeks 4 day	Control	142,2163	****	****		
6	4 weeks 7 day	Control	143,2837	****	****	****	
5	4 weeks 7 day	PheAla	156,0937	****	****	****	****
9	5 weeks 4 day	PheAla	171,3157		****	****	****
7	5 weeks 2 day	PheAla	173,9130			****	****
8	5 weeks 2 day	Control	185,5707				****

2.4 Comparison of homogeneous groups: control cultures and cultures fed with phenylalanine, in terms of furanocoumarins production.

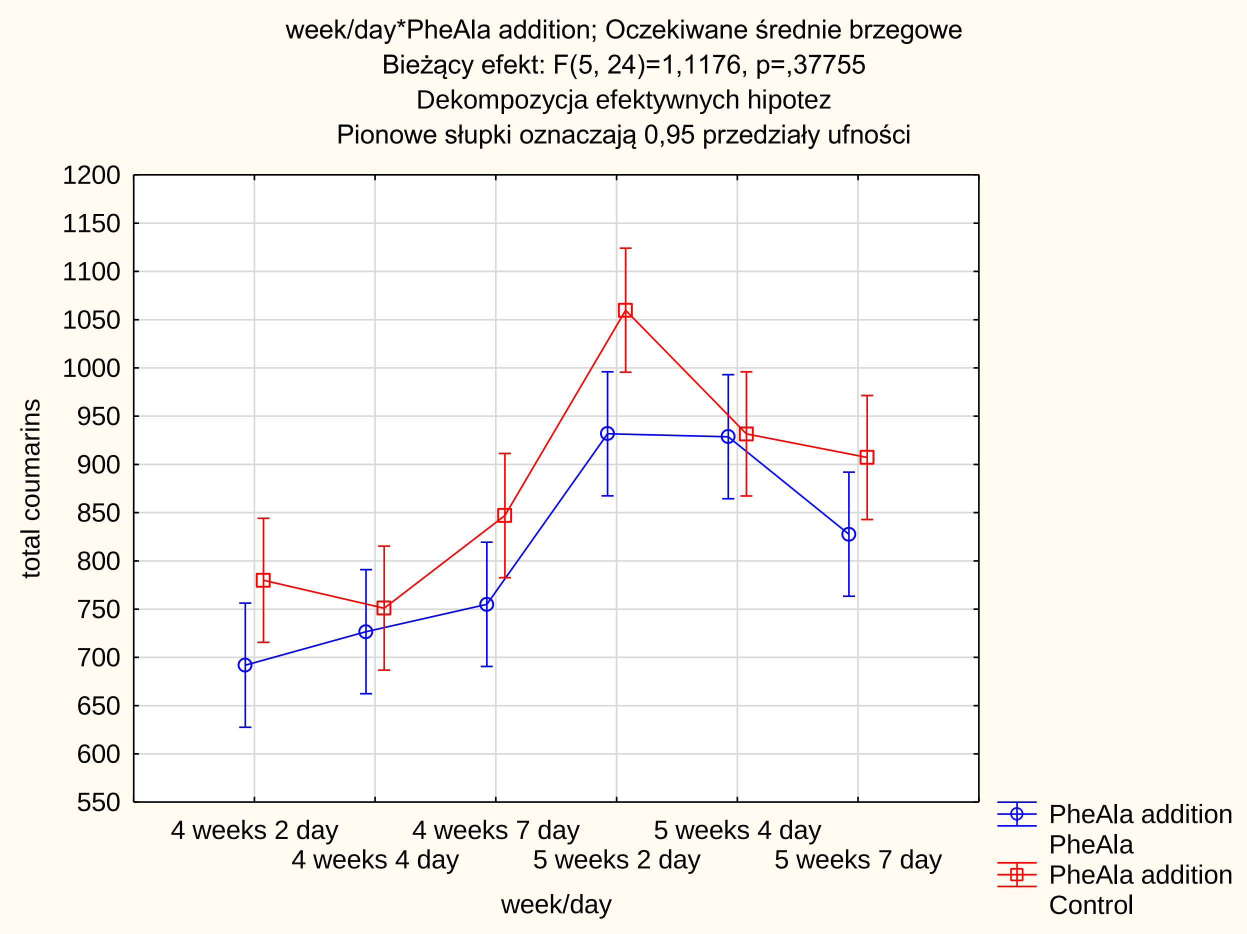

No.	Tukey test; total coumarins; homogenous groups; alpha = 0.05						
	week/day	PheAla addition	total coumarins	1	2	3	4
1	4 weeks 2 day	PheAla	691,904	****			
3	4 weeks 4 day	PheAla	726,631	****			
4	4 weeks 4 day	Control	751,044	****		****	
5	4 weeks 7 day	PheAla	754,967	****		****	
2	4 weeks 2 day	Control	779,898	****	****	****	
11	5 weeks 7 day	PheAla	827,671	****	****	****	
6	4 weeks 7 day	Control	847,013	****	****	****	
12	5 weeks 7 day	Control	907,222		****	****	****
9	5 weeks 4 day	PheAla	928,703		****		****
10	5 weeks 4 day	Control	931,637		****		****
7	5 weeks 2 day	PheAla	931,745		****		****
8	5 weeks 2 day	Control	1059,867				****
